# The DNA damage sensor ATM kinase interacts with the *p53 mRNA* and guides the DNA damage response pathway

**DOI:** 10.1186/s12943-024-01933-z

**Published:** 2024-01-23

**Authors:** Konstantinos Karakostis, Laurence Malbert-Colas, Aikaterini Thermou, Borek Vojtesek, Robin Fåhraeus

**Affiliations:** 1 Inserm UMRS1131, Institut de Génétique Moléculaire, Paris Cité Université, Hôpital St. Louis, Paris, France; 2 Institut de Biotecnologia I de Biomedicina, Universitat Autònoma de Barcelona, Bellaterra (Barcelona), Spain; 3 Research Centre for Applied Molecular Oncology (RECAMO), Masaryk Memorial Cancer Institute, Brno, Czech Republic; 4 Department of Medical Biosciences, Umeå University, Umeå, 90185 Sweden

**Keywords:** MDM2, Synonymous mutations, RNA secondary structure, Genotoxic stress, MRN complex, DNA Damage Sensing, Precision medicine

## Abstract

**Background:**

The ATM kinase constitutes a master regulatory hub of DNA damage and activates the p53 response pathway by phosphorylating the MDM2 protein, which develops an affinity for the *p53 mRNA* secondary structure. Disruption of this interaction prevents the activation of the nascent p53. The link of the MDM2 protein—*p53* mRNA interaction with the upstream DNA damage sensor ATM kinase and the role of the *p53 mRNA* in the DNA damage sensing mechanism, are still highly anticipated.

**Methods:**

The proximity ligation assay (PLA) has been extensively used to reveal the sub-cellular localisation of the protein—mRNA and protein–protein interactions. ELISA and co-immunoprecipitation confirmed the interactions in vitro and in cells.

**Results:**

This study provides a novel mechanism whereby the *p53 mRNA* interacts with the ATM kinase enzyme and shows that the L22L synonymous mutant, known to alter the secondary structure of the *p53* mRNA, prevents the interaction. The relevant mechanistic roles in the DNA Damage Sensing pathway, which is linked to downstream DNA damage response, are explored. Following DNA damage (double-stranded DNA breaks activating ATM), activated MDMX protein competes the ATM—*p53 mRNA* interaction and prevents the association of the *p53 mRNA* with NBS1 (MRN complex). These data also reveal the binding domains and the phosphorylation events on ATM that regulate the interaction and the trafficking of the complex to the cytoplasm.

**Conclusion:**

The presented model shows a novel interaction of ATM with the *p53* mRNA and describes the link between DNA Damage Sensing with the downstream p53 activation pathways; supporting the rising functional implications of synonymous mutations altering secondary mRNA structures.

**Supplementary Information:**

The online version contains supplementary material available at 10.1186/s12943-024-01933-z.

## Background

The DNA damage response pathway activated to repair DNA double-strand breaks, involves the recruitment of Ataxia-telangiectasia mutated (ATM) and the MRN complex (Mre11, Rad50, NBS1) to the DNA breaks sites and the induction of the DNA damage response (DDR) pathway [1]. The ATM kinase acts as a sensor of DNA damage governing downstream signaling pathways aiming to repair damages and maintain genome stability, including the DDR pathway. The DDR is initiated by auto-activation of ATM, leading to kinase-active monomers via acetylation modifications of the C-terminus and auto-phosphorylation at serine 1981 and inactivating mutations, such as the ATM(S2592C), are frequent in cancer samples [2]. A key downstream target of ATM is the p53 tumour suppressor that promotes cell cycle arrest and maintains genomic stability and cellular homeostasis. ATM activates p53 by phosphorylating the MDM2 E3 ubiquitin ligase at Ser395 [3] and its homologue, MDMX/4 at Ser403 [4, 5], which switch their activities from negative to positive regulators of p53 by forming interactions between their C-terminal RING domains with the *p53 mRNA,* leading to an increase in p53 protein synthesis, stabilisation and activation. The role of the *p53* mRNA was described by a model involving the export of the MDM2-interacting components (*p53* mRNA, RPL5, RPL11) to the cytoplasm and the formation of the ribosome that translates p53, allowing ATM to reach and phosphorylate the serine 15 of the nascent p53 peptide, thus stabilizing it and activating it towards the DDR [5, 6]. This model describes how ATM gains access and phosphorylates the serine 15 of the nascent p53 peptide substrate via MDM2 and emphasized the role of the *p53 mRNA* in the positive regulation of MDM2 towards p53. It also exposed how synonymous mutations (SMs) altering the *p53 mRNA* secondary structure have a direct negative effect on the stabilization and activation of p53 [6, 7], advancing evidence showing RNA structures uptaking roles in the damage-induced recruitment of repair factors to the DNA damage sites. However, the biological functions of SMs and of altered mRNA secondary structures in cell regulation and their clinical implementation remain under-investigated and poorly understood, urging the need for mechanistic studies. A single cancer patient-derived synonymous mutation at *p53* codon 22 (L22L) prevents the MDM2—*p53 mRNA* interaction and abolishes the MDM2-mediated activation of p53 following genotoxic stress [8]. This, together with the fact that the *p53* mRNA – MDM2 interaction evolved prior to the p53 – MDM2, protein – protein interaction, emphasizes the important role of the *p53 mRNA* secondary structure in governing p53 activity [7]. These pioneer results have contributed to break-through studies showing that certain single SMs trigger structural modifications of secondary mRNA structures, with direct effects on the translation and activation of the encoded proteins or/and deleterious carcinogenic effects [9–12]. In fact, SMs have functional consequences in over 50 diseases and drive natural selection and cancer development by impacting the activity of oncogenes and tumour suppressors, including mRNAs encoding TP53, KRAS and IGF-1, thus playing crucial roles in cellular regulation [6, 8, 10, 12, 13]. However, mechanistic studies such as the current one are anticipated for addressing the roles of the secondary mRNA structures in cell regulation and for exploring the involvement of the *p53 mRNA* – MDM2 interaction to the upstream global DNA damage Sensing signaling, mediated by the ATM kinase. This work provides insights on the signalling mechanism upstream of the ATM phosphorylation of p53 at serine 15, leading to the activation of p53 following DNA damage and does not supplant the existing pathway [4, 5].

## Methods

### Plasmids, recombinant proteins and cell lines

The p*cDNA* vector was used for eukaryotic expression and the p*ET28a* vector for bacterial expression. The p*ET28* constructs were introduced in BL.21 E. *coli* for the preparation of recombinant rFAT and rPI3K proteins. HA or FLAG tags were fused to the 5’ of the CDS as indicated and each cloning was confirmed by direct sequencing. Single nucleotide mutants were prepared using as template the p*cDNA-flag-atm.* The constructs and the priming oligonucleotides are outlined in Supplementary Methods (Supplementary Methods). The cell lines H1299 (human non-small cells lung cancer, not expressing p53 CRL-5803, ATCC), AT5-BIVA (ATM -/-) (ATM-deficient), A549 (human lung carcinoma cells, CCL-185 ATCC) and the human colorectal carcinoma cell line HCT116 were used. Cells were incubated, transfected and treated as performed previously and described in Supplementary Methods (Supplementary Methods). Briefly, cells were incubated at 37 °C, 5% CO_2_ in RPMI medium (for H1299) or DMEM medium (for AT5 or A549), supplemented with antibiotics, 2 mM L-glutamine (Gibco/Invitrogen) and 10% fetal serum (Hyclone). Cells were transfected with small amounts of DNA (a total of 100 ng/ml plasmid DNA). Depending on the experiment, cells were treated with either DMSO or 0.5 to 1 μΜ Doxorubicin (genotoxic stress) for 1 to 16 h. For the A549 and HCT116 cell lines used in coIP assays, see below, “RNA Binding assay (co-immunoprecipitation) section”.

### RNA Binding assay (co-immunoprecipitation)

For the CoIP assays of the endogenous *p53* mRNA with ATM on A549 and HCT116 cells, the rabbit anti-ATM pAb was used (ab10939, Abcam) and DNA damage was induced by either Doxorubicin (Doxo) 1 mM or Etoposide (Etopo) 50 μM; or Actinomycin D (ActD) 1.5 μM; or 1.5 μM Mitoxantrone (MXT), for 3 h. For the CoIP assay using H1299 cells transfected with p53 constructs and ATM-FLAG, the anti-FLAG antibody was used. The asterisks in the graphs represent the *P* values of one-tailed paired t-test, as follows: ‘n/s’ for *P* > 0.05; ‘*’ for *P* ≤ 0.05; ‘**’ for *P* ≤ 0.01; and ‘***’ for *P* ≤ 0.001. The error-bars represent the standard deviations of three independent experiments. The followed procedure is described in detail in the supplementary section (Supplementary Methods).

### Immunochemistry, Proximity Ligation Assay (PLA)

Cells were grown on sterilized a 24-well plate, transfected and fixed in 4% PFA for IF and PLA. After three washes with PBS for 10 min and then incubation with blocking buffer (3% BSA, 0.1% saponin in PBS), samples were incubated with primary antibodies for 2 h at RT. The used antibodies and the probes are outlined in Supplementary Methods (Supplementary Methods). Each sample was tested in triplicates and the PLA signal dots were counted by the ImageJ software. The values were analyzed using the GraphPad Prism 8 software (GraphPad Software, Boston, Massachusetts USA, www.graphpad.com). The asterisks in the graphs represent the *P* values of two-tailed unpaired t-test), as follows: ‘n/s’ for *P* > 0.05; ‘*’ for *P* ≤ 0.05; ‘**’ for *P* ≤ 0.01; and ‘***’ for *P* ≤ 0.001. The error-bars represent the standard deviations of three independent experiments.

### In vitro RNA–Protein (RPI) ELISA and Protein–Protein (PPI) sandwich ELISA

The biotinylated *p53 mRNA* was in vitro synthesised and 96-well plates were coated, blocked, incubated with the biotinylated *p53 mRNA* or proteins and washed, as described in Supplementary Methods (Supplementary Methods). Each sample was tested in triplicates in three independent experiments and the values were analyzed using GraphPad Prism 8 software. The asterisks in the graphs represent the *P* values of t-test, as follows: ‘n/s’ for *P* > 0.05; ‘*’ for *P* ≤ 0.05; ‘**’ for *P* ≤ 0.01; and ‘***’ for *P* ≤ 0.001. The error-bars represent the standard deviations of three independent experiments.

## Results

### The p53 mRNA interacts with ATM kinase in normal conditions, but following DNA damage activated MDMX competes for the p53 mRNA preventing its association with the NBS1 protein of the MRN complex

**Fig. 1:**
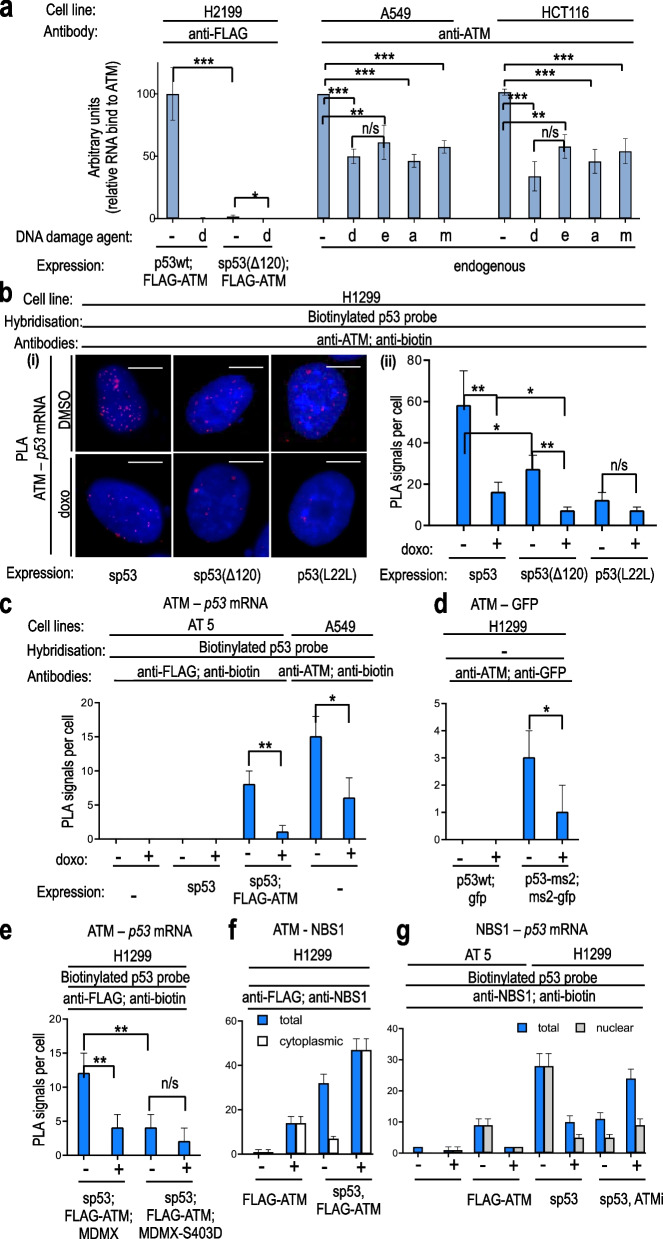
The ATM kinase interacts with the *p53 mRNA* in an MDM2-dependant and DNA damage dependant manner. **a** Co-immunoprecipitation coupled with qPCR (RNA binding) testing the ATM—*p53* mRNA interaction, of H1299 cells (p53-null) transfected with ATM (FLAG-ATM) and either p53wt or p53(Δ120) lacking the 5’CDS and *box-I* sequence, and of endogenous targets in A549 and HCT116 cells. Treatments include: “- “: dmso; “d”: doxorubicin; “e”: etoposide; “a”: actinomycin D; "m”: mitoxantrone. **b-i** Images of Proximity Ligation Assay (PLA) on H1299 cells, transfected with silenced p53 sequences of either wt, or p53(Δ120) lacking the 5’CDS and *box-I* sequence, or p53(L22L) expressing a modified secondary structure of the *box-I*. The y-axis represents the PLA signal per cell (amount of puncta per cell) (**b-ii**). **c** PLA on AT5 BiVA cells (ATM-null), transfected with combinations of ATM (FLAG-ATM) or silenced p53wt, as indicated; and on A549 cells expressing endogenous ATM and p53. **d** PLA on H1299 cells, transfected with either *p53wt* and *gfp* or *p53-ms2* and *ms2-gfp*. A PLA signal is exclusively seen with the *ms2* transfected constructs, using anti-ATM and anti-GFP antibodies, confirming that the ATM interacts with the *p53 mRNA*. **e** PLA on H1299 cells, transfected with silenced p53 and FLAG-ATM and either MDMX or the phosphor-mimetic mutant MDMX-403D that activates its interaction with the *p53 mRNA*. In normal conditions, the PLA signal is decreased in the presence of MDMX(403D), showing that the ATM-p53 *mRNA* interaction is competed by MDMX and that activated MDMX develops an affinity for the *p53 mRNA*. **f** PLA on H1299 cells, transfected with either FLAG-ATM or silenced p53 and FLAG-ATM, showing that the *p53 mRNA* promotes the association of ATM with NBS1 of the MRN complex in the nucleus during normal conditions and in the cytoplasm following DNA damage. **g** PLA on AT5 cells (ATM-null) and H1299 cells (p53-null) testing the effect of ATM activity on the NBS1—*p53 mRNA* interaction. ATM is required for the association of NBS1 with the *p53 mRNA* in the nucleus and inhibition of ATM activity prevents the association in the nucleus. The asterisks in the PLAs represent t-test *p* values of three independent experiments, as follows: ‘n/s’ for *P* > 0.05; ‘*’ for values *P* ≤ 0.05; ‘**’ for values *P* ≤ 0.01; and ‘***’ for values *P* ≤ 0.001. The statistical analyses of the PLAs are based on results obtained by at least 50 cells of each experiment. The cell lines, hybridization probes and antibodies used for each PLA are indicated

It was previously shown that following DNA damage, ATM phosphorylates MDM2 at Ser-395 facilitating the binding of MDM2 on the *p53 mRNA* that leads to the stabilization of the nascent p53 by ATM [6, 8]. We further tested the interplay between the ATM kinase, MDM2 and the *p53 mRNA* by testing if ATM also interacts with the *p53 mRNA*. We performed RNA co-immunoprecipitation (RNA CoIP) on H1299 whole cell extracts, expressing FLAG-tagged ATM and p53wt and we observed an ATM – *p53 mRNA* interaction under normal conditions, which is prevented following doxorubicin treatment (Fig. 1a). Similarly, endogenous targets on A549 and HCT116 cells showed a 50% decrease in the binding of the *p53* mRNA to ATM, when the cells were treated with doxorubicin or etoposide (Fig. 1a). These results show the activation of ATM, which is triggered by genotoxic agents, such as doxorubicin or etoposide or actinomycin D or mitoxantrone, significantly reduce the interaction of ATM with the *p53* mRNA. One-tailed paired t-tests were used to compare the means between two treatments on each cell type. One-Way ANOVA (Welch’s) analysis showed no significant variation among the different cell lines tested here, showing that in all the tested cell lines the ATM—*p53* mRNA interaction takes place under normal conditions while ATM is inactivated, and it is prevented following DNA damage (double stranded DNA breaks) that activates the ATM. Furthermore, the interaction was also prevented when we instead transfected the p53(Δ120) construct that lacks the + 1 to + 120 nt, and the conserved *box-1* sequence that controls the primary interaction site of MDM2 (Fig. 1a). These results show that 5’ terminal of the p53 coding sequence is required for binding ATM, similarly to MDM2. In order to identify the localisation of ATM interacting with the *p53 mRNA*, we employed the Proximity Ligation Assay (PLA). Following expression of a silent *p53 mRNA* construct (*sp53*) that lacks the first, second and third in-frame AUG initiation codons and does not express the p53 protein, we could detect nuclear PLA signals in H1299 cells under normal conditions using biotinylated DNA probes (nt 666 to 731) against the *p53 mRNA*, and a combination of anti-biotin and anti-ATM antibodies (Fig. 1b; Supplementary Fig. 1a; Supplementary Fig. 2a). This PLA signal was reduced by approximately 50% when we instead used the *p53(Δ120)* or the *p53(L22L)* mRNA constructs, which exhibits a poor affinity for MDM2 [6] as well as following doxorubicin treatment (Fig. 1b; Supplementary Fig. 1a). Additionally, we employed the AT5BIVA (ATM -/-) cell line, which is ATM-deficient, derived from an individual with ataxia-telangiectasia (AT). Using anti-FLAG and anti-biotin probes on AT5 cells (ATM null), no PLA signal between ATM and the *p53 mRNA* was observed but it was restored when exogenous ATM was introduced (Fig. 1c; Supplementary Fig. 1a). Furthermore, using a similar approach we observed the endogenous ATM—*p53 mRNA* interaction on A549 cells expressing wild type ATM and p53. This interaction was reduced following doxorubicin treatment (Fig. 1c; Supplementary Fig. 1a). We next took a different approach to test the ATM—*p53 mRNA* interaction by fusing the *ms2* binding sequence to the *p53 mRNA* and express this construct together with a MS2-GFP fusion protein (MS2-GFP). We could then observe the protein—protein PLA signal using anti-ATM together with anti-GFP antibodies, confirming that the PLA interaction is not due to technical artefacts related to the *p53 mRNA* probe (Fig. 1d; Supplementary Fig. 1b). Similarly to the ATM—*p53 mRNA* PLA signal, the ATM-GFP PLA signal was reduced following treatment with doxorubicin (Fig. 1d; Supplementary Fig. 1b). When we introduced the MDMX(S403D) construct, which mimics the phosphorylated MDMX(S403) and binds the nascent *p53 mRNA* in normal conditions [14], there were significantly fewer ATM – *p53 mRNA* PLA signals, showing that once MDMX is activated during the DNA damage response, it competes with ATM for the interaction with the *p53 mRNA*, regardless of the activation state of ATM [8] (Fig. 1e; Supplementary Fig. 1c).

These results indicate that the ATM—*p53 mRNA* interaction is linked to the upstream, global DNA damage sensing pathway, involving the interaction of ATM with components of the MRN complex. In order to test this hypothesis, we interrogated the interaction of NBS1 with ATM on H1299 cells, as the Nbs1 is necessary for ATM recruitment to MRN-bound DSBs and ATM activation. The NBS1 is expressed in the nucleus under normal conditions; and following treatment with doxorubicin it partially translocates to the cytoplasm (Fig. 1f; Supplementary Fig. 1d). The *p53* mRNA induced the ATM-NBS1 interaction under normal conditions in the nucleus, while following DNA damage and translocation of NBS1 to the cytoplasm, the *p53 mRNA* instead promoted the ATM-NBS1 interaction to the cytoplasm. Furthermore, we used AT5 (ATM null) and H1299 (p53 null) cells to test the role of ATM to the association of NBS1 with the *p53 mRNA*. RNA PLA experiments on the AT5 cells did not show an association of NBS1 with the *p53 mRNA* in the absence of ATM. After transfecting exogenous ATM (FLAG-ATM construct), this association was restored. These results clearly demonstrate that ATM is required for the NBS1—*p53 mRNA* association, strongly indicating that this association is mediated by ATM (Fig. 1g; Supplementary Fig. 1e). It is noted that these results show that the *p53* mRNA does not directly bind NBS1, it rather gets into association with NBS1, via the ATM kinase. This association takes place in the nucleus under normal conditions, while following doxorubicin treatment, it is prevented, indicating that upon DNA damage and ATM activation, the NBS1 no longer associates with ATM. Indeed, when we treated the H1299 cells with ATM inhibitors, the NBS1—*p53 mRNA* PLA signal was prevented in the nucleus showing that the association of NBS1 with the *p53 mRNA* is via ATM and the dynamic activation of ATM is required for the nuclear association (Fig. 1g; Supplementary Fig. 1e). These results show that the NBS1—ATM interaction is increased in the nucleus, while during DDR, it is exclusively cytoplasmic (Fig. 1f). Under normal conditions, the interaction of the ATM kinase with the NBS1 protein of the MRN complex in increased by the introduction of the *p53 mRNA* in the nucleus. Following activation of ATM (ie DNA damage) and loss of the ATM—*p53 mRNA* interaction, the NBS1 protein interacts with ATM at the cytoplasm, but it is no longer associated with the *p53 mRNA*.

### The FAT and the PI3K domains of ATM bind the p53 mRNA

**Fig. 2:**
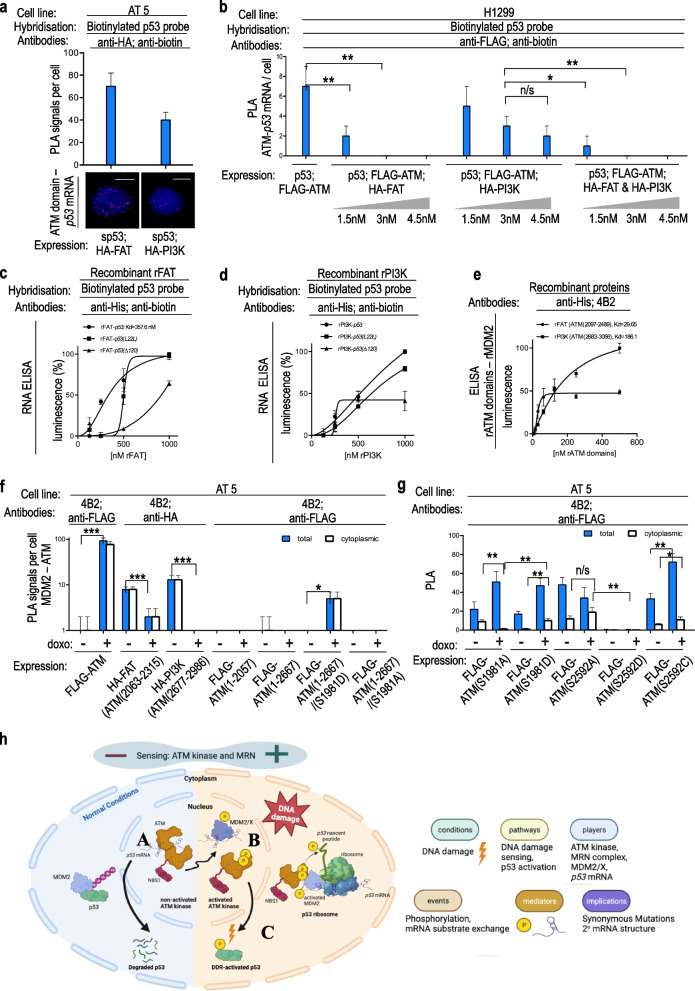
**a** PLA on AT5 BiVA cells (ATM-null), transfected with HA-FAT or HA-PI3K domains of ATM. PLA signal (amount of *puncta* per cell) shows that both domains independently bind the *p53 mRNA*, but the FAT domain binds it more prominently. Scale bars represent 10 μm. **b** PLA on H1299 cells (p53-null), transfected with FLAG-ATM and increasing amounts of HA-FAT or HA-PI3K or both (0, 1.5, 3.0, 4.5 nM). PLA signals show that both FAT and PI3K domains prevent the interaction of the full length ATM with the *p53 mRNA* in a constitutively manner. **c**, **d** Sandwich RNA ELISA using recombinant FAT domain (c) or PI3K domain (d) and in vitro synthesized *p53* mRNA (*p53wt* or *p53(L22L)* or *p53(Δp120)*), showing the kinetics of the interactions. The FAT domain shows an increased affinity for the *p53 mRNA* compared to the PI3K domain. **e** Sandwich ELISA using his-tagged recombinant FAT domain or PI3K domain proteins with recombinant MDM2 protein. The FAT domain more potently binds the MDM2 protein, compared to the PI3K domain. **f** PLA on AT5 BiVA cells, transfected with different constructs partially lacking domains of ATM, to test the interaction of MDM2 with ATM. **g** PLA on AT5 BiVA cells, transfected with different phosphomimetic or phospho-impaired ATM constructs at S1981 or S2592. PLA signals show that the ATM-MDM2 interaction is regulated by Ser-2592 and takes place on the FAT and the PI3K domains, whereas the trafficking of the ATM/MDM2 complex to the cytoplasm is regulated by ATM activity and via the phosphorylation of Ser-1981. The asterisks represent *P* values of three independent experiments, as follows: ‘n/s’ for *P* > 0.05; ‘*’ for values *P* ≤ 0.05; ‘**’ for values *P* ≤ 0.01; and ‘***’ for values *P* ≤ 0.001. The statistical analyses are based on results obtained by at least 50 cells of each experiment. The cell lines, hybridization probes and antibodies used for each PLA are indicated. **h** Model describing how the ATM kinase exchanges the *p53 mRNA* substrate with MDM2, ultimately leading to the phosphorylation of the nascent p53 peptide (p53-Ser-15) that activates p53 towards the DNA damage response

To determine the *p53 mRNA*-binding region of ATM, we expressed two HA-tagged domains of ATM, the FAT and the PI3K domains (Fig. 2a; Supplementary Fig. 2a; Supplementary Fig. 3a) and performed PLA experiments. We observed PLA signals for each domain with the *p53 mRNA* in AT5 cells under normal conditions. We obtained approximately 60% more PLA signals for the FAT domain interacting with the p53 *mRNA*, than for the PI3K domain (Fig. 2a). In order to test the specificity of the ATM—*p53 mRNA* interaction, we did competition PLA experiments expressing increasing amounts of either the FAT or the PI3K domains and probed the PLA signal of the full-length ATM—*p53 mRNA* interaction in H1299 cells. This resulted in a dose-dependent loss of the ATM – *p53 mRNA* PLA signal (Fig. 2b; Supplementary Fig. 2b; Supplementary Fig. 3b). The FAT domain was more efficient in preventing the full-length ATM—*p53 mRNA* interaction compared to the PI3K domain and even completely prevented this interaction from the concentration of 3 nM, when the PI3K domain only decreased it by 50%. Interestingly, when we co-expressed in total 3 nM of the FAT and the PI3K domains together, the full-length ATM—*p53 mRNA* interaction was completely prevented. Furthermore, we expressed in vitro recombinant versions of the FAT and the PI3K domains separately (Supplementary Fig. 2c; Supplementary Fig. 3a) and used them in sandwich ELISA. The rFAT domain, showed an affinity for the in vitro synthesized *p53wt* mRNA (Kd 357.6 nM) as well as for *p53(L22L) mRNA* and *p53(*Δ*120) mRNA* (Fig. 2c). The rPI3K domain similarly showed an affinity for the *p53 mRNA*. Each domain showed different kinetics (Fig. 2d). The affinity for a short synthetic RNA (+ 1 to + 120) to ATM was very low (data not shown). This is also observed for MDM2 and MDMX that require a structure of the *p53 mRNA* involving (+ 1 to + 120) formed during RNA synthesis, implicating that ATM, similarly to MDM2 and MDMX, recognizes a specific structure on the *p53 mRNA*. These data collectively show that under normal conditions ATM, via the FAT and the PI3K domains, directly binds a structure on the *p53 mRNA* that depends on the 5’ coding sequence, similarly to MDMX and MDM2.

### Phosphorylation events on ATM act synergistically to control the interaction of the FAT and PI3K domains with MDM2 for the trafficking of this complex to the cytoplasm

Following DNA damage, MDM2 interacts with ATM and translocate to the cytoplasm to promote the synthesis of p53. Having shown that the FAT and PI3K domains of ATM bind the *p53 mRNA*, we set up to test if the same domains also interact with MDM2. We initially performed the sandwich ELISA method using recombinant versions of MDM2, rFAT and rPI3K to test the direct interaction between them. The FAT domain showed an affinity of Kd 30 nM for MDM2, while the PI3K a Kd 186 nM (Fig. 2e; Supplementary Fig. 2c). Each protein exhibited diverse kinetics suggesting that they interact with different epitopes on MDM2. We then determined by PLA the sub-cellular localization of each interaction. Whereas the full length ATM interacts with MDM2 in the nucleus following doxorubicin treatment but not under normal conditions (Fig. 2f; Supplementary Fig. 2a; Supplementary Fig. 4), the FAT and the PI3K domains were sufficient to bind MDM2 in the nucleus under normal conditions (Fig. 2f; Supplementary Fig. 4). However these domains interacted poorly following doxorubicin treatment. Removal of the C-terminus containing the FAT and the PI3K domains, ATM(1–2057), completely abolished the PLA signal. Similarly, adding the FAT domain but not the PI3K domain, ATM(1–2667), did not significantly restore the interaction under normal or DDR conditions, suggesting the FAT domain is under conformational restrain in this construct. However, the interaction of ATM(1–2667) with MDM2 following doxorubicin treatment was partially restored by inserting the phosphomimetic S1981D mutation, but not the S1981A (Fig. 2f; Supplementary Fig. 4). These results suggest that the MDM2 epitopes, located within the FAT and the PI3K domains of ATM, are regulated by phosphorylation events, thus promoting conformational change in the folding of ATM that allows the exposure of these MDM2 epitopes.

We then tested mutations at the phosphorylation sites Ser-1981 and Ser-2592 that are known to regulate the oligomerization and activity of ATM. When we mutated ATM at Ser-1981 to Ala, we could observe ATM(S1981A) – MDM2 PLA signals in the nucleus under normal conditions (Fig. 2g). In line with previous data showing the trafficking of the ATM/MDM2 complex to the cytoplasm following DNA damage, this signal was mainly observed in the nuclear compartment following doxorubicin treatment. When we instead introduced the ATM(S1981D) phosphomimetic mutation, we observed ATM(S1981D) – MDM2 PLA signals exclusively in the nucleus under normal conditions, while doxorubicin treatment resulted in both nuclear and cytoplasmic association (Fig. 2g; Supplementary Fig. 2a; Supplementary Fig. 4). These results show that the trafficking of the ATM/MDM2 to the cytoplasm is regulated via ATM(S1981) and that the phosphorylation induced by DNA damage is necessary but not sufficient for the translocation to the cytoplasm. When we instead mutated Ser-2592 to Ala (ATM(S2592A)) we observed a constitutive interaction with MDM2 that was not affected by doxorubicin treatment, as it was partially present in the cytoplasm, regardless of the DNA damage conditions. Importantly, the ATM(S2592D) did not interact with MDM2 under any conditions. Furthermore, the kinase-inactive cancer-derived mutant ATM(S2592C) interacted with MDM2 predominantly in the nuclear compartment, showing that this mutation perturbs the trafficking resulting from the dynamics of the interaction (Fig. 2g; Supplementary Fig. 4). These data collectively show that the interaction between ATM and MDM2 takes place on the FAT and the PI3K domains and is regulated via phosphorylation on Ser-2592, whereas the trafficking of the ATM/MDM2 complex to the cytoplasm is regulated by ATM activity and via the phosphorylation of Ser-1981. Additionally, diverse ATM phosphorylation events, act dynamically together to control the exposure of the MDM2 epitopes on ATM and the trafficking of the complex (Fig. 2h). The model shows that (A) Under normal conditions, The ATM homodimer is inactivated and interacts with the p53 mRNA in the nucleus. MDM2 binds and catalyses the poly-ubiquitination of p53, targeting it for degradation via the 26S proteasomal pathway in the cytoplasm. (B) Following DNA damage, ATM is activated by auto-phosphorylation and phosphorylates MDM2 at Ser395 promoting it’s binding to the p53 mRNA. (C) The MDM2-p53 mRNA interactions leads to the formation of the ATM-MDM2-RPL5-RPL11-*p53* mRNA complex, according to the previously described model [6]. This association facilitates the export of the complex to the cytoplasm and the formation of polysomes expressing p53. ATM phosphorylates the nascent p53 peptide at Ser-15, thus preventing MDM2 from binding the emerging p53 peptide. This model explains how MDM2 gains access and interacts with the *p53* mRNA while it gets phosphorylated by the ATM kinase, contributing to the previous downstream model describing how MDM2 can stimulate the translation of p53 without degrading the newly synthesized p53 protein and how a single synonymous mutation can affect the stability of the encoded protein.

## Discussion

DNA damage response in human cells is primarily driven by the ATM kinase that constitutes a hub of interactions, phosphorylating more than 700 substrates [15]. In response to genotoxic stress, the MRN complex is recruited to DNA breaks and activates ATM via NBS1 [1]. ATM interactions with MR and with the NBS1, are shown to be required for its activation and mutants close to the Ser-1981 site of ATM (ie. T1985E/S1987D/S1988D) decrease the binding to both MRN and MR [16]. NBS1 plays an important role ATM activation [1]. DNA damage results to the activation of ATM by inducing the monomerization of the inactive ATM dimer, following auto-phosphorylation of ATM(S1981), which in turn activates p53. Here, we show that inactivated ATM interacts with the *p53 mRNA* secondary structure. Employing DNA damage agents, we measured by coIP-RNA the decrease of the interaction in several cell lines, both on transfected targets (H1299) and on endogenous targets (A549 and HCT116 cell lines). Results clearly show that the ATM-*p53* mRNA interaction takes place under normal conditions and it is prevented following ATM activation resulting from exposure to DNA damage agents. Comparatively, all tested drugs prevent the endogenous ATM-*p53* mRNA interaction by about 50% **(**Fig. 1A**)**. The ATM constitutes a hub of interactions and it is linked to activities of the MRN complex and H2AX at sites of double-stranded DNA damage. In line with this work, it is known that during DNA damage, the inhibition of RNA polymerase II leads to the prevention of transcription and thus the pre-existing pools of mRNAs are suggested to play a key role in repair mechanisms. It is also documented that RNA binding proteins constitute ATM substrates, coupling the DDR pathway with the modulation of RNA metabolism and ATM was shown to suppress the mRNA binding of the transcription/export complex THOC5. As such, it could be hypothesised that a pool of *p53* mRNAs interacting with ATM at the nucleus during normal conditions, may serve as reserve that is used for the downstream MDM2-dependant p53 activation upon DNA damage.

We show that ATM activation upon S1981 phosphorylation, abolishes the interaction. This event is shown to be in coordination with the phosphorylation activity of ATM targeting MDM2. Similarly to the effect of the *p53* mRNA secondary structure on binding ATM (shown here) or MDM2 (shown previously [6]), functional roles of mRNA molecules binding to signalling proteins have started to unravel, and accumulating findings show that SMs can alter the secondary structures and the expression levels of the encoded proteins [6, 10], implicating diseases. In cancer, the impact on the mRNA secondary structure and the protein levels, has only been documented for *TP53, KRAS* and *IGF-1* mRNAs [6, 10, 12], but SMs retaining the capacity to alter the secondary mRNA structure, are associated with tumors and are currently estimated to 470 representing 6–8% of all driver mutations occurring due to single nucleotide substitutions [10, 13]. Their significant occurrence frequencies and effects have been especially shown for genes of the RAS family [12]; NOTCH1 and p53 [17], all of which constitute components of highly mutated pathways in several cancers.

Focusing on the ATM-p53-MDM2/X model, MDM2/X is known to interact with the *p53 mRNA* [6] and with the *E2F1*, *Rb* and *Xiap* mRNAs [14], while the interplay between p53 and MDM2 is well conserved in the evolution [18] and the *p53 mRNA*—MDM2 interaction is detected in pre-vertebrates [7]. Once phosphorylated by ATM, MDM2(S395) mediates the translocation of the *p53 mRNA* and the precursor complex RPL5-RPL11, components of the 5S RNP assembly, to the cytoplasm, promoting the synthesis of p53. It also results to the translocation of ATM to the nascent p53 promoting its phosphorylation at p53(S15) that prevents the binding of p53 to MDM2 and its degradation via the E3 ligase activity, thus activating p53 towards the DDR [6]. However, (i) the mechanism whereby MDM2 targets the *p53 mRNA* to promote the activation of p53; and (ii) the link to the upstream DNA damage sensing pathway and the activation of ATM, was not explored. The signalling link of the p53 activation pathway to the upstream global DNA damage sensing pathway is highly anticipated to expand the conceptual findings of the roles of the secondary mRNA structures in gene regulation and pathway activation.

Here we advance previous mechanistic findings on DNA damage and the impact of the secondary *p53* mRNA structure by investigating its role in the global DNA damage sensing pathway. We show for the first time that inactivated ATM interacts with the *p53 mRNA*, both in vitro and in cells. This interaction takes place at the nucleus so a hypothesised translation-preventing effect of the ATM-*p53* mRNA interaction during normal conditions would require an additional mechanism mediating the translocation of the *p53* mRNA to the cytoplasm. Following DNA damage and ATM activation, MDMX competes ATM for binding the *p53 mRNA*, strongly indicating a mechanism whereby MDMX gains access to the *p53* mRNA via ATM. This interaction has been documented but the underlying mechanism describing how it is targeted was unknown. Indeed, MDMX competes ATM for binding the *p53 mRNA* following DNA damage and the FAT and PI3K domains competed the full-length ATM in binding the *p53 mRNA* **(**Fig. 2b**)**. In addition, the p53(L22L) synonymous mutation, which is known to prevent the MDM2—*p53 mRNA* interaction during DDR, also prevents the interaction of the *p53 mRNA* with the ATM kinase under normal conditions, showing a conserved vital role of the mRNA secondary structure to its interaction with components of the p53 pathway. It is possible that the described mechanism maybe similarly employed to regulate translation of other mRNAs and RNA binding factors that similarly interact with the ATM kinase.

Results also demonstrate that conformational changes induced by the activation of ATM implicating the phosphorylation sites S1981, which is used as a marker of activated ATM, and the breast cancer breast cancer Ser-2592, mutation, regulate the exposure of the FAT and the PI3K domains of ATM. These domains directly interact with both the *p53 mRNA* and with the MDM2 protein. According to the literature, ATM forms a framework on which various proteins bind and structural studies have demonstrated that ATM interchangeably forms an open dimer with a limited intermolecular interface and a tightly packed closed dimer with a larger interface. It was proposed that the binding of a partner on ATM could stabilise the closed dimer [19]. Moreover, the FAT domain of ATM wraps around the kinase domain and the Ser-1981 phosphorylation enables ATM to remain associated with sites of double-strand DNA breaks [19]. In line with these structural aspects of ATM, we show here that both the FAT and PI3K domains interact with the *p53 mRNA* under normal conditions, while the activation of ATM by phosphorylation on S1981 following doxorubicin treatment promotes the nucleus-cytoplasmic shuttling of the ATM—MDM2 complex **(**Fig. 2h**)**. In addition, even though the ATM(S2592) site is not reported to effect on ATM activation, it regulates the dynamics of the MDM2-ATM interaction in normal and DNA damage conditions. We show that the phosphomimetic S2592D mutation leads to the abrogation of the interaction, while the S2592A (phospho-inhibiting mutation), leads to constant interaction with MDM2. Similarly, the kinase-inactive cancer-derived mutant ATM(S2592C) interacts with MDM2 under all conditions and mainly in the nucleus **(**Fig. 2g; Supplementary Fig. 4). Both ATM(S1981) and ATM(S2592) sites have been shown to be phosphorylated following DNA damage and their dynamic phosphorylation is shown to be required for the cytoplasmic translocation of the ATM-MDM2 complex, as both phospho-mimetic and phospho-inhibiting mutants, sequestered the complex to the nucleus. Interestingly, the sub-cellular localisation of ATM has been correlated with the activation of distinct downstream signaling pathways beyond DNA damage, as for example the ROS-induced TSC2 pathway resulting in mTORC1 repression and autophagy, which is regulated by cytoplasmic ATM [20]. Cytoplasmic ATM was also shown to modulate synaptic functions in neurons [21], while roles in oxidative stress and metabolic pathways, associated with cell migration and increased metastatic potential, have been proposed [22]. Perspective studies will shed light onto potential roles of the interaction of ATM with mRNAs and the phosphorylations on S1981 and S2592, regulating its cellular compartmentalization. The investigation of the effect of ATM-derived patient mutations including SMs altering the *p53* mRNA secondary structures is highly anticipated to address the clinical and translational implications of this mechanism and of SMs altering the secondary mRNA structures.

## Conclusions

This study provides a novel mechanism through which the secondary structure of the *p53 mRNA*, interacts with the ATM kinase of the DNA damage sensing pathway. These results expand the previous model describing the role of the *p53 mRNA* in the MDM2-dependant activation of p53 following DNA damage, by linking the upstream ATM pathway (ie the DNA damage sensing proteins: ATM and components of the MRN complex). These data support a model explaining how ATM gains access to target and phosphorylate MDM2, while MDM2 gets access to the *p53* mRNA during DNA damage. The exchange of the *p53* mRNA substrate from ATM to MDM2 is coordinated with the activation of ATM following DNA damage and the phosphorylations of MDM2/X by ATM. In situ PLAs show the sub-cellular localization of the interacting partners and biochemical assays confirm that MDMX competes the ATM—*p53 mRNA* interaction and prevents the association of the *p53 mRNA* with the NBS1 protein of the MRN complex, via ATM. In addition, structural data employing recombinant mutants and PLA and ELISA assays reveal that phosphorylation events on ATM act synergistically to control the interaction of the FAT and PI3K domains of ATM with MDM2 and its trafficking to the cytoplasm. Our results show the mechanism whereby synonymous mutations altering the secondary structure of the *p53 mRNA* prevent the DNA damage sensing and response pathways (Fig. 2h). Altogether, these findings advance the mechanistic aspects of the roles or SMs and demonstrate the role of the secondary structure of the *p53 mRNA* in the signalling, linking the DNA damage sensing pathway with the DDR pathway. The presented model further supports that such synonymous mutations could modulate DNA damage responses and provide with the mechanistic background to further investigate their potential translational value. The significance of the ATM-*p53 mRNA* interaction is that via the described mechanism, the *p53* mRNA reaches MDM2 following DNA damage, enhancing its translation. This research is of broad interest to the DNA-repair and structural biology field, expanding previous mechanistic studies of cell signalling leading to the activation of the p53 pathway and introducing novel concepts of regulatory roles of enzymes (ie ATM) interacting with mRNA.

### Supplementary Information

**Additional file 1: Supplementary Methods**.

**Additional file file 2: Supplementary Figure 1.** Representative microscopy images of the PLA assays corresponding to Fig. 1: (a) Images corresponding to Fig. 1b and c. (b) Images corresponding to Fig. 1d. (c) Images corresponding to Fig. 1e. (d) Images corresponding to Fig. 1f. (e) Images corresponding to Fig. 1g. Scale bars represent 10 μm. The cell lines, hybridization probes and antibodies used for each PLA are indicated.

**Additional file file 3: Supplementary Figure 2.** (a) Immunofluorescence controls for protein expression/location and antibody cross reactivity, on H1299 cells transfected with each ATM construct, using anti-FLAG or anti-HA antibodies. Expression constructs are indicated. Both normal (DMSO) and doxorubicin conditions are shown. DAPI is stained in Blue and Specific Antibodies are stained in Red. Scale bars represent 10 μm. (b) Immunofluorescence controls of NBS1. (c) SDS Gel electrophoresis showing the bacterial expression of recombinant rFAT and rPI3K domains of ATM (left) and western blot using anti-His antibody (right).

**Additional file file 4: Supplementary Figure 3.** (a) Graphical Illustration of the domains of the ATM kinase: (i) The TAN, FAT, PI3K and FATC domains are mapped on the 3056aa long ATM sequence and the phosphorylation sites Ser-1981 and Ser-2592 are noted. (ii) CDSs of the FAT and PI3K domains, used for pcDNA3 clonings for cellular expression. (iii) CDSs of extended regions involving either the FAT or the PI3K domain, in between the Ser-1981 and Ser 2592, used in pET28 clonings for recombinant bacterial expression. (iv) truncated ATM(1-2057) construct cloned in pcDNA3 vectors includes the S1981 activation site but excludes the FAT and the PI3K domains and the S2592 site. (v) truncated ATM(1-2667) construct includes both phosphorylation sites and the FAT domain but excludes the PI3K domain and the C’terminal. (b) Representative microscopy images of the PLA assays corresponding to Fig. 2b. Scale bars represent 10 μm. The cell lines, hybridization probes and antibodies used for each PLA are indicated. Dapi (in Blue) stains the nucleus and the PLA signal is stained Red.

**Additional file file 5: Supplementary Figure 4.** Representative PLA microscopy images corresponding to Fig. 2f. DAPI is stained in Blue and PLA dots are stained in red. Scale bars represent 10 μm.

## Data Availability

The data supporting the conclusions of this article are included within the article.

## Abbreviations

ActD: Actinomycin D
ATM: Ataxia–telangiectasia mutated
BSA: Bovine serum albumin
coIP: Co-immunoprecipitation
DAPI: 4′,6-Diamidino-2-phenylindole
DDR: DNA damage response
Doxo: Doxorubicin
DSBs: DNA double-strand breaks
ELISA: Enzyme-linked immunosorbent assay
Etopo: Etoposide
FAT: FRAP-ATM-TRRAP
IF: Immunofluorescence
MRN: Mre11, Rad50, NBS1
MXT: Mitoxantrone
PBS: Phosphate-buffered saline
PFA: Paraformaldehyde
PLA: Proximity Ligation Assay
PI3K: Phosphatidylinositol 3-kinase
qPCR: Quantitative polymerase chain reaction
RBPs: RNA binding proteins
rFAT: Recombinant protein domain FAT
rPI3K: Recombinant protein domain PI3K
RT: Room temperature
SM: Synonymous mutation
TAN: Tel1/ATM N-terminal motif
