# Supplementary material for: The DNA damage sensor ATM kinase interacts with the p53 mRNA and guides the DNA damage response pathway
Source: Mol Cancer. 2024 Jan 23;23:21. doi: 10.1186/s12943-024-01933-z (PMC10804554; doi:10.1186/s12943-024-01933-z)
Supplement: Supplementary file 1 — Additional file 1: Supplementary Methods. [file 12943_2024_1933_MOESM1_ESM.pdf]

Supplementary Methods

Plasmids and recombinant proteins

Plasmids were cloned using the p*cDNA* vector for eukaryotic expression and the p*ET28a* vector for bacterial expression. On occasion, HA or FLAG tags were fused to the 5' of the CDS as indicated. Each cloning was confirmed by direct sequencing. The constructs and the oligonucleotides (5'-3') are the following:

p*ET28-FAT(ATM(2097-2489):* TTAGGATCCATGGAACTTCATTACCAAGCA and CATTACTCGAGCTAGGAACAAAGTCGGAATAC*;*

p*ET28-PI3K(ATM(2683-3056):* TTAGAATTCATGATACAGTCATTTAAAGCAGAA and CATTACTCGAGCTACATGGTCCAGTCAAAGAG*;*

p*cDNA-FAT(ATM(2063-2315*) (HA):
TTACTCGAGATGTACCCATACGATGTTCCAGATTACGCTGGAATCATTCAGGCCTTGC and CCGGGGCCCTCACATTTGCTTGAGAATACTCAGG*;*

p*cDNA-PI3K(ATM(2677-2986)* (HA):
TTACTCGAGATGTACCCATACGATGTTCCAGATTACGCTGGAAATCTGGTGACTATACAG TC and CCGGGGCCCTCATGCATTCAGAGTAGGGTGAAG*;*

p*cDNA-flag-atm:* TTAGTCGACATGAGTCTAGTACTTAATGATCTGCTTATCTGCTG and CATTACTCGAGTCACACCCAAGCTTTCCATCCTG*;*

Subsequently, point mutants were prepared using as template the p*cDNA-flag-atm* The constructs and the oligonucleotides (5'-3') are the following:

p*cDNA-atm(S1981D):*
GAAGTCTTGCATTTGAAGAAGGAGACCAGAGTACAACTATTTCTAGCTTGAG*;*

p*cDNA-atm(S1981A):*
GAAGTCTTGCATTTGAAGAAGGAGCCCAGAGTACAACTATTTCTAGCTTGAG*;*

p*cDNA-atm(S2592D):*
GAATAACTAAAAATGTGCCTAAACAAAGCGATCAGCTTGATGAGGATCGAACAG*;*

p*cDNA-atm(S2592A):*
GAATAACTAAAAATGTGCCTAAACAAAGCGCTCAGCTTGATGAGGATCGAACAG*;*

p*cDNA-atm(S2592C):*
GAATAACTAAAAATGTGCCTAAACAAAGCTGTCAGCTTGATGAGGATCGAACAG*;*

p*cDNA-atm(1-2057):*
CATATGACCTCGAAACAGCAATCCCCTGATCAACACGCCAGGCAGGAATC*;*

p*cDNA-atm(1-2667):*

GAAGATGTTGTTGTCCCTACTATGTAAATTAAGGTGGACCACACAGGAG*;*

p*cDNA-atm(1-2667)/(S1981A):*

GAAGTCTTGCATTTGAAGAAGGAGCCCAGAGTACAACTATTTCTAGCTTGAG and GAAGATGTTGTTGTCCCTACTATGTAAATTAAGGTGGACCACACAGGAG.

The plasmids p*cDNA-p53* and p*cDNA-mdm2* containing the full-length CDSs, have been described previously [1, 2]. The p*ET28* constructs were introduced in BL21 E. *coli* for the preparation of recombinant rFAT and rPI3K proteins. Over-expression was induced by 1 mM IPTG at 28 ^{o}C for 6 h. Each protein from the bacterial lysate was purified in 50 mM Tris and 100 mM NaCl pH 8 following a standard protocol using affinity nickel chromatography and the AKTA pump (Amersham), and then dialysis. The purity of each protein was confirmed by gel electrophoresis and western blotting and the proteins were used for the preparation of specific antibodies in mice, rabbits and in chickens. The recombinant MDM2 protein used in the assays was previously prepared [3].

Transient transfection of cell lines

The cell lines H1299 (human non-small cells lung cancer, not expressing p53 CRL-5803, ATCC), AT5 [4-7] (not expressing ATM) and A549 [1] (human lung carcinoma cells, CCL-185 ATCC) were used. Each cell line was tested authenticated by PCR and was periodically tested for mycoplasma infection. Cells were incubated at 37 °C, 5% CO_2 in RPMI medium (for H1299) or DMEM medium (for AT5 or A549), supplemented with antibiotics, 2 mM L-glutamine (Gibco/ Invitrogen) and 10% fetal serum (Hyclone). Cells were transfected with small amounts of DNA (a total of 100 ng/ml plasmid DNA). In the compared samples, the cells were transfected with the same transfection mix and the total p*cDNA3* concentration was equilibrated. The comparisons were exclusively made in samples of individual experiments using the same cell line and run under the same conditions. The expression of individual constructs was determined using IH. Depending on the experiment, cells were treated with either DMSO or 0.5 to 1 μM Doxorubicin (Sigma) for 1 to 16h, or with 15 μM ATM inhibitor KU55933 (Abcam ab120637) for 16h. They were subsequently either fixed in 4% PFA and used in immunohistochemistry or PLA or cross-linked by irradiation with 0.15 J/cm2 total energy of 365 nm UV light, then stored at -80 ^{o}C before analysis in western blotting or co-immunoprecipitation assays (CoIP).

RNA Binding assay (co-immunoprecipitation)

Transfected H1299 cells were cultured in RPMI medium as described above. A549 and HCT116 cells were cultured in DMEM medium supplemented with 10% fetal bovine serum (FBS), 2 mM L-glutamine, 100 U/mL penicillin, and 100 µg/mL streptomycin and were seeded in 10cm plates (1x106 cells/plate). For cell treatments with DNA damaging agents, the following treatments were separately performed in parallel with DMSO - treated counterpart samples: Doxorubicin (Doxo, Sigma) 1mM or Etoposide (Etopo, E1383, Sigma) 50µM; or Actinomycin D (ActD, Merck, A9415) 1.5µM; or 1.5 µM Mitoxantrone (MXT, Merck, M6545), for 3h at 37 °C. Confluent cells were collected after trypsin treatment and washed twice with 1X PBS (Gibco). Cells were suspended in 500 µl of Polysomal lysis buffer (100 mM KCl; 1M $MgCl_2$; 50 mM HEPES pH 7.4; 0.1% Igepal and 1X protease inhibitor cocktail Roche) containing 1mM DTT (Invitrogen) and 0.1U/µl RNAse Out (Invitrogen). Cell lysis was performed by a series of vortex followed by 30 min incubation on ice. After lysis cells were centrifuged at 4 °C for 20 min at 13000g, the supernatant was quantified by Bradford, and the same protein quantity was aliquoted and adjusted to the same final volume by adding Polysomal Lysis Buffer and RNAse Out. The lysates were pre-cleaned by adding 50µl of G-coated sepharose beads (cytiva) with 5µl of Rabbit serum (DAKO) and were incubated at 4 °C for 30min on a rotating wheel. After a fast centrifuge 2min at 8000g, the cleared lysates were kept, and 100µl were used for Total RNA fraction, by adding 900µl TRIzol (Invitrogen) and freezing at -80°C. The rest of the cleared lysates were incubated with 60µl of G-coated sepharose beads and 1µg anti-rabbit ATM antibody (ab10939, Abcam) overnight at 4 °C on a rotating wheel. In the case of H1299 cells transfected with FLAG-ATM, a FLAG tag Ab was used (2368S Cell Signalling). After centrifuge 2 min at 8000g the supernatant was removed and the beads were resuspended in Polysomal Lysis Buffer, 25% of the suspended beads were eluted in 2X SDS loading buffer and analyzed by western blot against anti-rabbit ATM antibody (or anti-FLAG antibody). Then the beads were washed 4 times with 450µl of combination of diluted buffers: W1) 1X Binding buffer (50 mM Tris pH 7.5; 150 mM NaCl); W2) BB1X / PBS 4/2 volumes; W3) BB1X / PBS 2/4 volumes and W4) BB1X / PBS 1/5 volumes. The washes were followed by Proteinase K treatment, by adding 100µl of BB1X / PBS 1/5, 0.1% SDS, RNaseOUT, and 30ng Proteinase K (Sigma) to the beads and incubating for 30 minutes at 55°C. Both the bound and total RNA fractions were then extracted and purified using TRIzol protocol (Invitrogen). cDNA synthesis was carried out using the Moloney murine leukemia virus M-MLV reverse transcriptase and Oligo(dT)12-18 primer (Life technologies). qPCR was performed using the StepOne real-time PCR system (Applied Biosystems) with Perfecta SYBR Green FastMix (Quanta Biosciences) using

primers against p53 (Forward: 5’ TGGGCTTCTTGCATTCTG 3’ and Reverse: 5’ GCTGTGACTGCTTGTAGATGGC 3’). We performed an absolute qPCR relative to the standard curve and the binding results were expressed as a ratio between the bound and the total RNA. The values were analyzed using the GraphPad Prism 8 software (GraphPad Software, Boston, Massachusetts USA, www.graphpad.com). The asterisks in the graphs represent the p values of one-tailed paired t-test of three independent experiments, as follows: ‘n/s’ for $P > 0.05$; ‘*’ for $P \leq 0.05$; ‘**’ for $P \leq 0.01$; and ‘***’ for $P \leq 0.001$. Anova statistics testing the effect of both the cell line factor (ie: A549 or HCT116) and the treatment factor (ie: either without drug (DMSO) or one of the DNA damaging agents: Doxo, Etopo, ActD, MXT) gave a p value $p<0.0001$, while Anova lacking the DMSO values, that compares only the DNA damaging agents, had a marginally significant p-value of $p=0.0166$, which can be attributed to the treatment conditions of each drug. Anova also showed no significant variation among the cell lines ($p=0.1113$).

Immunochemistry and the Proximity Ligation Assay (PLA)

Cells were grown on sterilized glass slides in a 24-well plate, transfected and fixed in 4% PFA for IF and PLA. After three washes with PBS for 10 min and then incubation with blocking buffer (3% BSA, 0.1% saponin in PBS), samples were incubated with primary antibodies for 2h at RT. The antibodies used were: the DO-1 mouse antibody recognising the N-terminal of p53 (in house [8]); the CM-1 rabbit antibody cross-reacting with p53 (in house [9]); the 4B2 anti-MDM2 mouse (in house [10]). Anti-MDM2 antibody was also purchased by Abcam: ab87134; rabbit). Anti-FLAG tag Abs were purchased by Cell Signalling: 2368S (rabbit) and by Abcam: ab18230 (mouse). Also, the anti-ATM Ab (Abcam, ab10939) and the anti-p95/NBS1 (Cell Signalling, p95/NBS1 (D6J5I) Rabbit mAb 14956) were purchased. All Abs were used in a dilution of 1:200. For IF detection, cells were incubated with Alexa-488-conjugated (Invitrogen) anti-rabbit Ab and Alexa Fluor-633-conjugated (Invitrogen) anti-mouse Ab. For the PLA, the PLA RED kit (Sigma) was used and the method described previously was followed [11-13]. For the RNA – protein interaction PLA (RPI-PLA), fixed cells were first hybridized with the 5’ biotin-tagged p53 probe with 0.5 ng/ul of the probe: ‘5- AAAAAACCCATGCAGGAACTGTTACACATGTAGTTGTAGTGGATGG TGGTACAGTCAGAGCCAACCTCAGG -3’, at 50 °C for 18 h. Sense probes used as negative controls did not label the cells. The labelling of the nucleus was done with DAPI. Images were captured using 640 nm filters on standard microscope; or by confocal microscopy when higher resolution was needed.. Each sample was tested in triplicates and the PLA signal dots were counted by the ImageJ software. For each sample, the counted cytoplasmic signals were localized in

individual cells, as observed by visible-light microscopy, during the capturing of each image. DAPI staining was used to determine nuclear signals and for each sample, we counted dots in 50 cells randomly selected during the imaging. This procedure was repeated in at least three independent experiments. As a transfection control, cells were co-transfected with GFP. In case transfection was required for the assay, the non-transfected cells were excluded from the statistical analyses. The values were analyzed using the GraphPad Prism 8 software. The asterisks in the graphs represent the two-tailed unpaired t-test P values of three independent experiments, as follows: 'n/s' for $P > 0.05$; '*' for $P \leq 0.05$; '**' for $P \leq 0.01$; and '***' for $P \leq 0.001$.

***In vitro* RNA-Protein (RPI) ELISA and Protein-Protein (PPI) sandwich ELISA**

The *p53 mRNA* was previously in vitro transcribed with the T7 mMessage mMachine kit (Ambion) using the p*cDNA-p53* plasmid as a template, purified with the RNeasy micro kit (Qiagen), then biotinylated using the RNA 3' End Biotinylation Kit (Thermo), following the instructions of the manufacturers. 96-well plates were coated overnight at 4°C with 100 µg/ml streptavidin (Biolabs, N7021S) in 0.1 M $NaHCO_3$, then blocked for 1h at RT in PBS with 3% BSA and 0.1 µg/ml streptavidin, then incubated for 1h at 37°C with a mixture of 0.5 pmol of biotinylated *p53* RNA with increasing concentrations of ATM protein (0 – 100 nM) in binding buffer (50 mM Tris pH 7.5, 150 mM NaCl, 20 µg/ml yeast tRNA and 0.2 mg/ml BSA). After washing, the plates were incubated for 1h at RT with the 6His mAB/HRP conjugate (1:1,000, Clontech) and luminescence was measured by the ECL medium. The p53 *mRNA* – MDM2 interaction was tested in parallel as a positive control. For the protein-protein sandwich ELISA, the protocol described by Abcam was followed. The DO-1; 4B2; and anti-His primary antibodies were used, with the corresponding secondary anti-IgG HRP antibodies. Each sample was tested in triplicates in three independent experiments and the values were analyzed using GraphPad Prism 8 software. The asterisks in the graphs represent the t-test P values of three independent experiments, as follows: 'n/s' for $P > 0.05$; '*' for $P \leq 0.05$; '**' for $P \leq 0.01$; and '***' for $P \leq 0.001$.

References

1. Bourougaa K, Naski N, Boularan C, Mlynarczyk C, Candeias MM, Marullo S, Fahraeus R: Endoplasmic reticulum stress induces G2 cell-cycle arrest via mRNA translation of the p53 isoform p53/47. *Mol Cell* 2010, 38:78-88.
2. Malbert-Colas L, Ponnuswamy A, Olivares-Illana V, Tournillon AS, Naski N, Fahraeus R: HDMX folds the nascent p53 mRNA following activation by the ATM kinase. *Mol Cell* 2014, 54:500-511.
3. Gajjar M, Candeias Marco M, Malbert-Colas L, Mazars A, Fujita J, Olivares-Illana V, Fåhraeus R: The p53 mRNA-Mdm2 Interaction Controls Mdm2 Nuclear Trafficking and Is Required for p53 Activation following DNA Damage. *Cancer Cell* 2012, 21:25-35.
4. Komatsu K, Matsuura S, Tauchi H, Endo S, Kodama S, Smeets D, Weemaes C, Oshimura M: The gene for Nijmegen breakage syndrome (V2) is not located on chromosome 11. *Am J Hum Genet* 1996, 58:885-888.
5. Matsuura S, Weemaes C, Smeets D, Takami H, Kondo N, Sakamoto S, Yano N, Nakamura A, Tauchi H, Endo S, et al: Genetic mapping using microcell-mediated chromosome transfer suggests a locus for Nijmegen breakage syndrome at chromosome 8q21-24. *Am J Hum Genet* 1997, 60:1487-1494.
6. Cheema AK, Timofeeva O, Varghese R, Dimtchev A, Shiekh K, Shulaev V, Suy S, Collins S, Ressom H, Jung M, Dritschilo A: Integrated analysis of ATM mediated gene and protein expression impacting cellular metabolism. *J Proteome Res* 2011, 10:2651-2657.
7. Zhao L, Chen H, Zhan YQ, Li CY, Ge CH, Zhang JH, Wang XH, Yu M, Yang XM: Serine 249 phosphorylation by ATM protein kinase regulates hepatocyte nuclear factor-1alpha transactivation. *Biochim Biophys Acta* 2014, 1839:604-620.
8. Vojtesek B, Bartek J, Midgley CA, Lane DP: An immunochemical analysis of the human nuclear phosphoprotein p53. New monoclonal antibodies and epitope mapping using recombinant p53. *J Immunol Methods* 1992, 151:237-244.
9. Midgley CA, Fisher CJ, Bartek J, Vojtesek B, Lane D, Barnes DM: Analysis of p53 expression in human tumours: an antibody raised against human p53 expressed in Escherichia coli. *J Cell Sci* 1992, 101 (Pt 1):183-189.
10. Chen J, Marechal V, Levine AJ: Mapping of the p53 and mdm-2 interaction domains. *Mol Cell Biol* 1993, 13:4107-4114.
11. Weibrecht I, Lundin E, Kiflemariam S, Mignardi M, Grundberg I, Larsson C, Koos B, Nilsson M, Soderberg O: In situ detection of individual mRNA molecules and protein complexes or post-translational modifications using padlock probes combined with the in situ proximity ligation assay. *Nat Protoc* 2013, 8:355-372.
12. Koos B, Andersson L, Clausson CM, Grannas K, Klaesson A, Cane G, Soderberg O: Analysis of protein interactions in situ by proximity ligation assays. *Curr Top Microbiol Immunol* 2014, 377:111-126.
13. Karakostis K, Lopez I, Pena-Balderas AM, Fahareus R, Olivares-Illana V: Molecular and Biochemical Techniques for Deciphering p53-MDM2 Regulatory Mechanisms. *Biomolecules* 2020, 11.
