# Supplementary figures and images for: The DNA damage sensor ATM kinase interacts with the p53 mRNA and guides the DNA damage response pathway
Source: Mol Cancer. 2024 Jan 23;23:21. doi: 10.1186/s12943-024-01933-z (PMC10804554; doi:10.1186/s12943-024-01933-z)

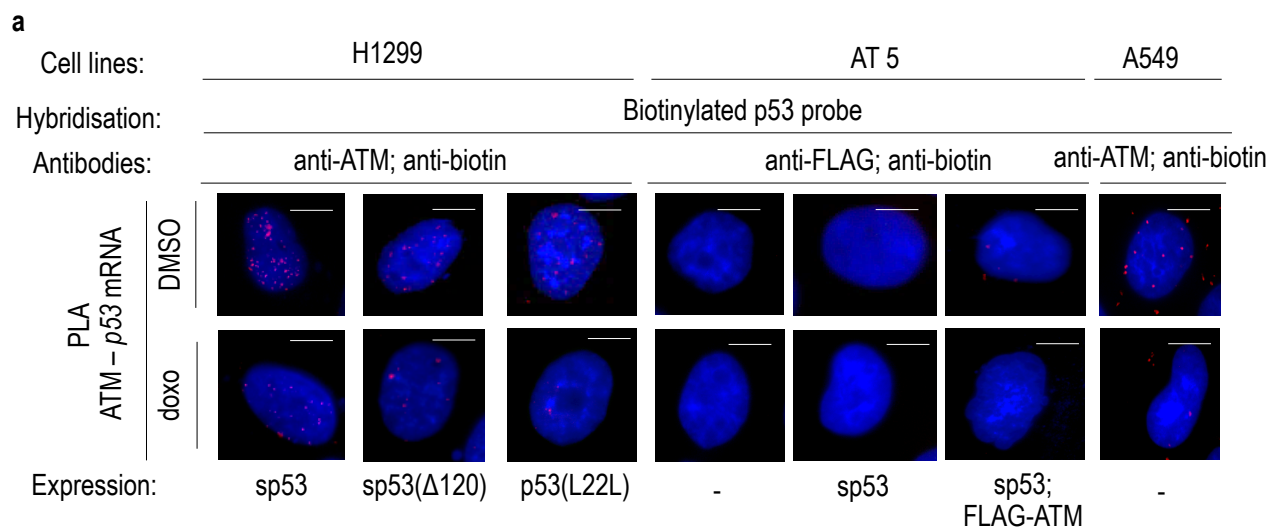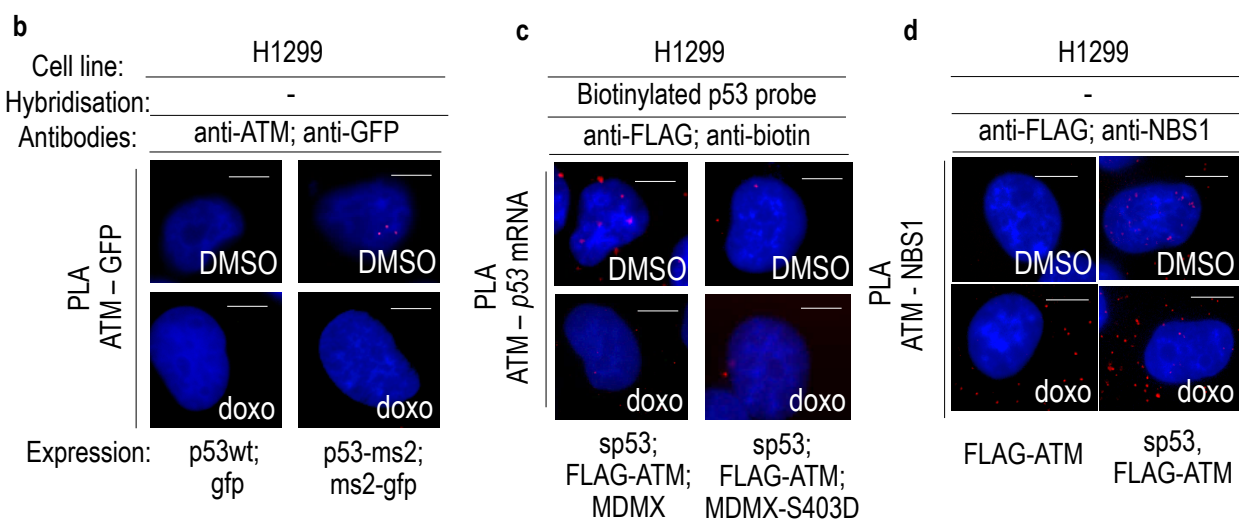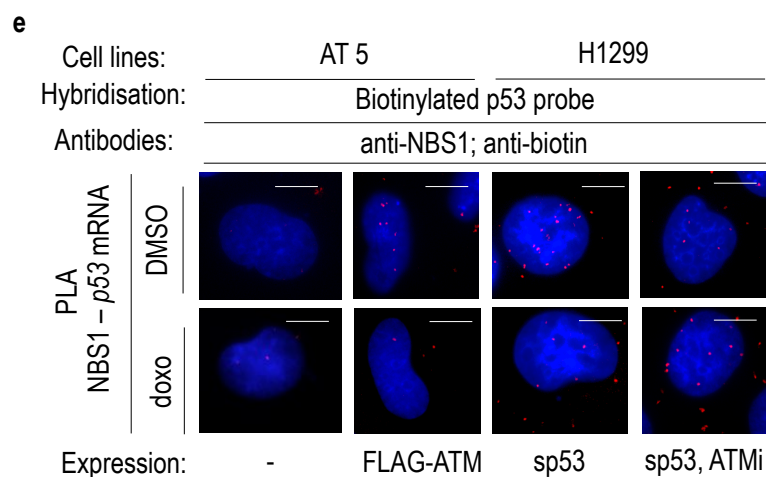

Supplement: Supplementary file 2 — Additional file file 2: Supplementary Figure 1. Representative microscopy images of the PLA assays corresponding to Fig. 1: (a) Images corresponding to Fig. 1b and c. (b) Images corresponding to Fig. 1d. (c) Images corresponding to Fig. 1e. (d) Images corresponding to Fig. 1f. (e) Images corresponding to Fig. 1g. Scale bars represent 10 μm. The cell lines, hybridization probes and antibodies used for each PLA are indicated. [file 12943_2024_1933_MOESM2_ESM.pdf]

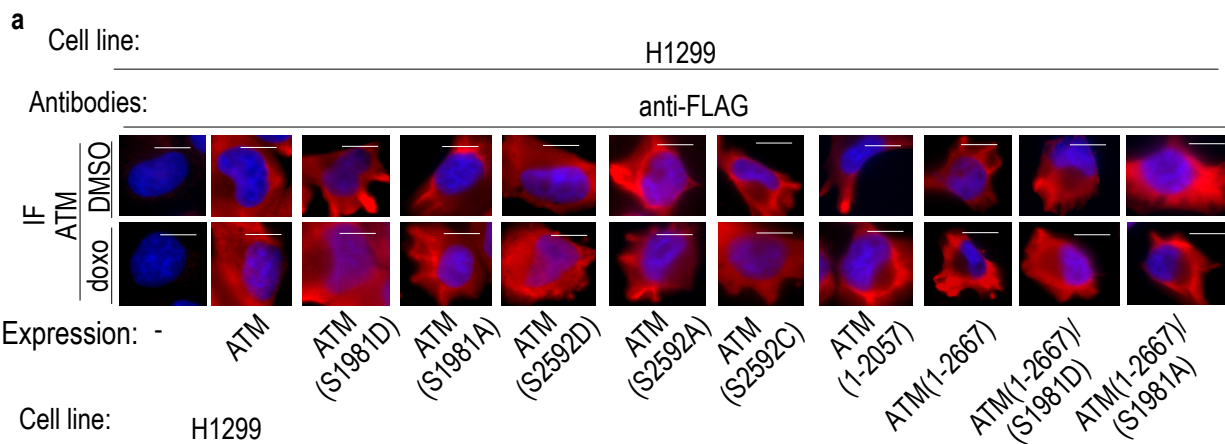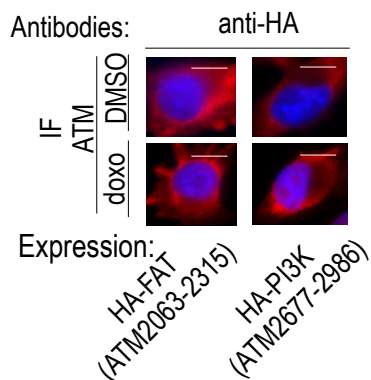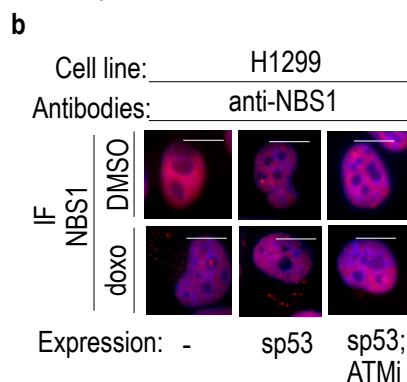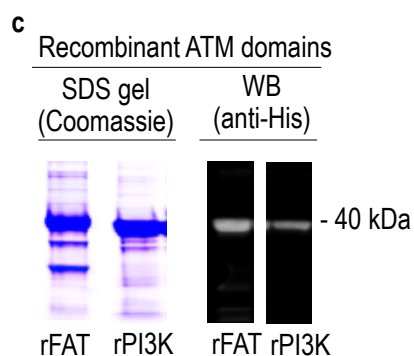

Supplement: Supplementary file 3 — Additional file file 3: Supplementary Figure 2. (a) Immunofluorescence controls for protein expression/location and antibody cross reactivity, on H1299 cells transfected with each ATM construct, using anti-FLAG or anti-HA antibodies. Expression constructs are indicated. Both normal (DMSO) and doxorubicin conditions are shown. DAPI is stained in Blue and Specific Antibodies are stained in Red. Scale bars represent 10 μm. (b) Immunofluorescence controls of NBS1. (c) SDS Gel electrophoresis showing the bacterial expression of recombinant rFAT and rPI3K domains of ATM (left) and western blot using anti-His antibody (right). [file 12943_2024_1933_MOESM3_ESM.pdf]

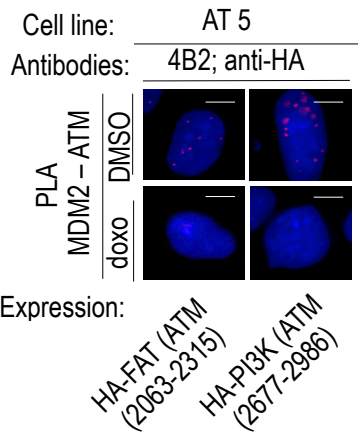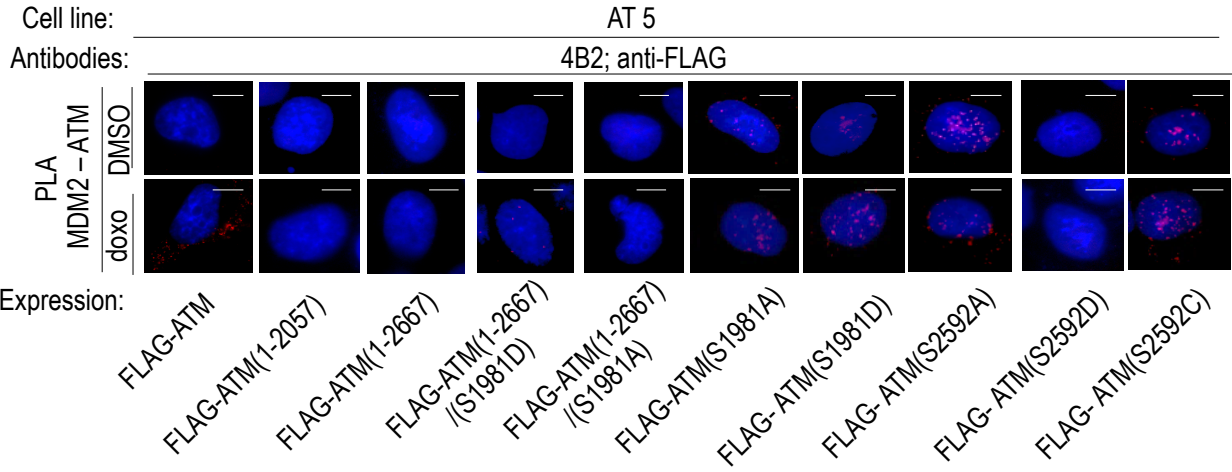

Supplement: Supplementary file 5 — Additional file file 5: Supplementary Figure 4. Representative PLA microscopy images corresponding to Fig. 2f. DAPI is stained in Blue and PLA dots are stained in red. Scale bars represent 10 μm. [file 12943_2024_1933_MOESM5_ESM.pdf]
