# Supplementary material for: The DNA damage sensor ATM kinase interacts with the p53 mRNA and guides the DNA damage response pathway
Source: Mol Cancer. 2024 Jan 23;23:21. doi: 10.1186/s12943-024-01933-z (PMC10804554; doi:10.1186/s12943-024-01933-z)
Supplement: Supplementary file 4 — Additional file file 4: Supplementary Figure 3. (a) Graphical Illustration of the domains of the ATM kinase: (i) The TAN, FAT, PI3K and FATC domains are mapped on the 3056aa long ATM sequence and the phosphorylation sites Ser-1981 and Ser-2592 are noted. (ii) CDSs of the FAT and PI3K domains, used for pcDNA3 clonings for cellular expression. (iii) CDSs of extended regions involving either the FAT or the PI3K domain, in between the Ser-1981 and Ser 2592, used in pET28 clonings for recombinant bacterial expression. (iv) truncated ATM(1-2057) construct cloned in pcDNA3 vectors includes the S1981 activation site but excludes the FAT and the PI3K domains and the S2592 site. (v) truncated ATM(1-2667) construct includes both phosphorylation sites and the FAT domain but excludes the PI3K domain and the C’terminal. (b) Representative microscopy images of the PLA assays corresponding to Fig. 2b. Scale bars represent 10 μm. The cell lines, hybridization probes and antibodies used for each PLA are indicated. Dapi (in Blue) stains the nucleus and the PLA signal is stained Red. [file 12943_2024_1933_MOESM4_ESM.pdf]

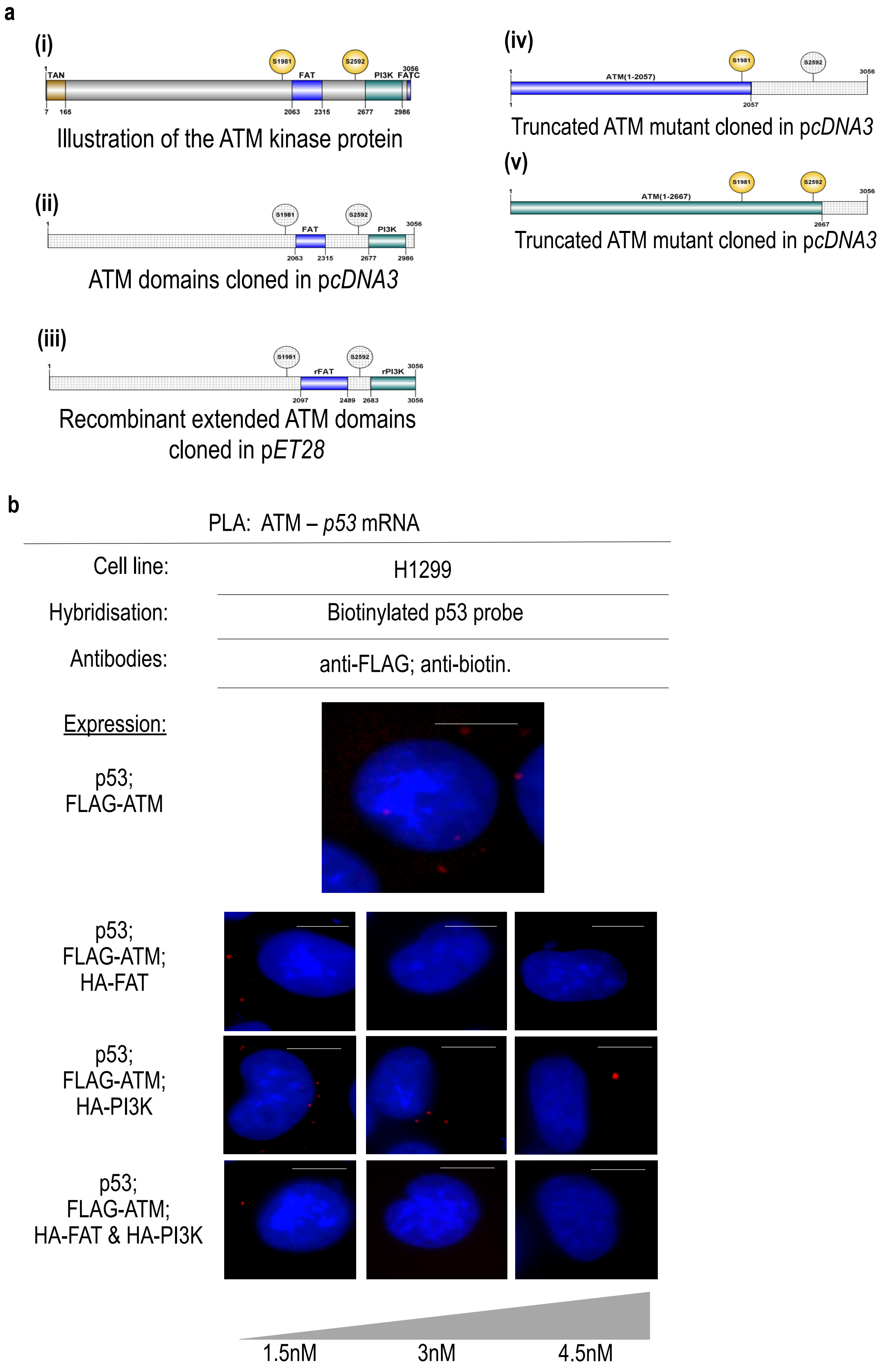
a
(i)
S1981
S2592
TAN
FAT
PI3K
FATC
1
3056
7
165
2063
2315
2677
2986
Illustration of the ATM kinase protein
(ii)
S1981
S2592
FAT
PI3K
3056
2063
2315
2677
2986
ATM domains cloned in pcDNA3
(iii)
S1981
S2592
rFAT
rPI3K
3056
2097
2489
2683
3056
Recombinant extended ATM domains cloned in pET28
(iv)
ATM(1-2057)
S1981
S2592
3056
2057
Truncated ATM mutant cloned in pcDNA3
(v)
ATM(1-2667)
S1981
S2592
3056
2667
Truncated ATM mutant cloned in pcDNA3
b
PLA: ATM – p53 mRNA
Cell line: H1299
Hybridisation: Biotinylated p53 probe
Antibodies: anti-FLAG; anti-biotin.
Expression:
p53; FLAG-ATM
p53; FLAG-ATM; HA-FAT
p53; FLAG-ATM; HA-PI3K
p53; FLAG-ATM; HA-FAT & HA-PI3K
1.5nM
3nM
4.5nM
